# Effectiveness of marker training for detection dogs

**DOI:** 10.3389/fvets.2025.1538452

**Published:** 2025-02-21

**Authors:** Lucia Lazarowski, Bart Rogers, Courtney Collins-Pisano, Sarah Krichbaum, Michael Handley, Jordan G. Smith, Paul Waggoner

**Affiliations:** ^1^Canine Performance Sciences, College of Veterinary Medicine, Auburn University, Auburn, AL, United States; ^2^Department of Anatomy, Physiology, and Pharmacology, College of Veterinary Medicine, Auburn University, Auburn, AL, United States; ^3^Department of Psychological Sciences, Auburn University, Auburn, AL, United States

**Keywords:** detection dogs, training, clicker training, conditioned reinforcer, canine, learning

## Abstract

Training detection dogs to alert to an odor requires precision in the timing and delivery of stimulus presentations in order to condition a strong association between odor and reward and to train a desired alert behavior that communicates the presence and location of the odor source. Marker training, in which a signal that predicts a reward is used to deliver immediate feedback for a correct response and bridge the delay between the desired behavior and reward, is a popular technique in the animal training industry. However, the application of marker training to detection dog training has not been examined, and empirical evidence of the purported benefits of marker training in general is lacking. The current study evaluated the effectiveness of marker training for odor detection learning and performance. Candidate detection dogs (*n* = 28) were trained to detect and alert to a target odor either with or without the use of a clicker as a marker (*n* = 14 per group). Effectiveness of marker training was assessed by comparing rate of learning the odor discrimination and the alert response, detection accuracy and topography of the alert behavior in an odor discrimination test, generalization of learned behavior from the odor recognition setting to a novel context (i.e., open-area operational searches), and resistance to extinction. Compared to dogs trained with the reward only, dogs trained with the marker as a signal for reward completed the training phase in fewer trials, performed the alert response more accurately in the odor recognition test, indicated the location of the odor source more precisely in the operational searches, and exhibited greater resistance to extinction when the primary reward for a correct response was withheld. These results provide evidence supporting the effectiveness of markers in animal training, and demonstrate benefits specific to the challenges commonly faced in detection dog training.

## 1 Introduction

The process of training detection dogs to locate and alert to a target odor involves conditioning an association between the odor and a reward (e.g., food or toy) as well as shaping a behavioral indication or “alert” to communicate the presence and location of the odor to the handler. Training methods vary widely (1, 2), and factors related to the timing and delivery of odor and/or reward can facilitate or hinder training time significantly (3). These variables have been well studied and described in the animal learning literature but have not been examined systematically in the application of detection dog training.

One variable of critical importance during initial conditioning of the odor-reward association is timing of stimulus presentations (3). The shorter the delay between odor and reward, the more rapid the conditioning. Traditional odor detection training typically includes odor–reward pairing by simultaneously presenting the odor and reward in close physical proximity (4). For example, dogs are given a play object (such as a towel, pipe, or toy) that contains the scent of the target odor so that the dog encounters the odor while playing with the object, thereby associating the odor with play. Other methods involve co-locating the reward (e.g., a ball or toy) and target odor in a box so that the dog encounters the two stimuli together when sniffing the box. Timing is also important in training the alert response, with shorter delays between the dog performing the desired indication behavior (e.g., sitting) and receiving the reinforcer resulting in better learning. Traditionally, an emphasis is typically placed on “rewarding at source” by delivering the reward to the dog directly at the source of the odor (e.g., tossing the ball so that it lands on or near the odor while the dog is at source). These methods strive to minimize the temporal delay and physical distance between the odor and the reward to strengthen the association; however, such practices can be problematic for several reasons. For one, physically pairing a target substance with the reward can lead to cross-contamination of odors which can alter the scent picture learned by the dog. Further, if the odor of the reward is stronger and more salient than the target odor, especially in the case of low vapor pressure odors or trace amounts, the odor of the reward can overshadow the odor of the target material, interfering with learning (3). In the case of explosives detection, delivering the reward at the source of the odor can pose hazards to the dog or handler when training with volatile explosives. Additionally, rewarding at source introduces inherent delays and necessary trainer involvement that can lead to reinforcement of undesirable behaviors. For example, immediately rewarding a dog upon detection of or response to the odor requires precision and speed in physically delivering the reward to the dog, often necessitating that the individual rewarding the dog be in close physical proximity. This often leads to the dog learning to associate other cues with the reward, such as the trainer’s body language, which can inadvertently become the discriminative stimulus to which the dog responds rather than the odor, and can lead to reinforcement of anticipatory behaviors such as looking in the direction of the trainer [known as sign tracking (5)]. Increasing the distance between the trainer and the dog minimizes such cueing, but the delay between response and reinforcement is necessarily increased and can lead to unintentional reinforcement of undesirable behaviors that occur in between the desired response and receipt of reward, as well as weakening the odor-reward association.

Marker training is a popular method in animal training in which a signal, typically auditory (e.g., a whistle, tone, click, or verbal cue), that has been previously associated with an established reinforcer is used to mark the exact moment a behavior is performed correctly (6). Because of the marker’s previously established association as a predictor of reward, it can serve as a conditioned reinforcer (also referred to as secondary reinforcer) of the behavior, and can bridge the temporal gap between the behavior and reward thus providing more immediate feedback to the animal (5–7). Use of a marker as a reward-predicting signal is considered to result in improved learning (54), especially when immediate delivery of the reward is not feasible due to the animal and trainer’s position or distance from each other (6). While the use of markers is widely embraced in the animal training industry, including zoos, aquariums, shelters, and training facilities, its adoption in detection canine training has been less common (8). Additionally, despite its widespread use and presumed benefits, experimental examinations of the effectiveness of marker training have found little evidence of its advantage over training without the use of markers (9–12). Its benefits, however, may depend on the nature of the target behavior or the training setting, and therefore may not have been evident in past studies due to methodological factors. In particular, researchers have suggested that marker training may be most effective in applied settings, especially when delivery of reinforcement is logistically difficult (11).

Given the challenges in timing of odor-reward presentations in detection dog training, marker training may be a valuable method because it: (1) provides a clear association between the odor and reward without potential confusion or contamination of the odors of the reward and the target of detection, (2) captures behavior immediately and precisely resulting in more efficient training of the desired alert response, (3) allows the dog to work at a distance from the handler thereby reducing handler dependence, influence, and unintentional cueing, and (4) allows the dog to be physically rewarded at a distance from the target if necessary. However, while studies have utilized markers in training dogs and other species to detect various odors (13–17), the effectiveness of marker training for such purposes has not been directly examined. The goal of this study was to assess whether training dogs to perform an odor detection task using a marker results in better training outcomes compared to training with a primary reward only. Candidate detection dogs that had yet to begin odor detection training were divided into two groups and were trained to perform a typical odor detection task either with or without the use of a marker. Dogs were trained using a standardized protocol to identify a target odor among a lineup of non-target odors and perform an alert response to indicate its location. Given the complexity of the task and the behavioral requirements of the target response compared to previous studies, we hypothesized that differences between groups would be observed. Specifically, we predicted that marker-trained dogs would complete training in fewer trials and demonstrate better performance compared to dogs trained with primary reinforcement only.

Marker training has historically been viewed with skepticism in the working dog industry, considered by trainers to not be beneficial and potentially counterproductive (8). One concern is that an emphasis on odor discrimination and performing the alert response in an artificial, contrived setting can result in context-specific obedience behavior and diminish more natural hunting behavior (18). Therefore, following training in an odor recognition setting, dogs in the current study were tested for transfer of the learned behavior to a novel context (i.e., open area search) to determine whether marker training facilitated or hindered generalization of the learned behavior from the specific context in which it was trained to a new and more naturalistic search context. For detection canines, transfer of learning from the initial training environment to an operational search environment would lead to valuable reductions in training time. We hypothesized that the performance of marker-trained dogs in the context generalization search test would be equivalent to, or better than, dogs trained with primary reinforcement only.

Another potential advantage of markers used as conditioned reinforcers is their ability to maintain a trained behavior in the absence of a primary reward, an effect which has been demonstrated experimentally with companion dogs trained to perform an operant response (12). For detection canines, in situations where delivering the primary reward is impractical or hazardous, the ability to maintain the behavior using a conditioned reinforcer could be valuable in preventing extinction. Thus, the final phase of the study involved a session in which primary reinforcement was withheld for all dogs. We hypothesized that providing the marker for correct responses without the primary reward would result in greater resistance to extinction compared to dogs receiving neither.

## 2 Methods

### 2.1 Subjects

Twenty-eight purpose-bred candidate detection dogs (Labrador retrievers, 10 F/18 M) from the Auburn University College of Veterinary Medicine (AUCVM) Canine Performance Sciences (CPS) detection dog breeding program participated in the study. All dogs were born and raised in the program and underwent standardized puppy development and socialization beginning at birth [for details, see (19)]. The current study took place as the dogs entered the final phase in the program at approximately 10 months of age, at which point they were still reproductively intact. Aside from searching for toys, none of the dogs had prior training in formal odor detection or in performing the target alert behavior in the study. Dogs were individually housed in indoor/outdoor runs in the kennel complex of the AUCVM during the study period. All activities were approved and monitored by the Auburn University Institutional Animal and Care Use Committee (IACUC # 2021–3943). The AUCVM is an Association for Assessment and Accreditation of Laboratory Animal Care International (AAALAC) accredited facility. After dogs concluded data collection for the study, they completed the remainder of the CPS training program until approximately 12–14 months of age at which point they were either placed in service in an operational detection role (*n* = 17), or retained in the AUCVM CPS program as a breeder (*n* = 6) or for non-operational detection work (*n* = 5).

### 2.2 General procedures

Dogs were assigned to one of two experimental groups at the onset of the study. To minimize pre-existing differences between the groups in motivational factors that could influence performance in the study, groups were balanced based on scores on a routine internal behavioral assessment conducted the week prior to the start of the study (see (20) for details on the assessment). Specifically, measures related to reward engagement were used and included “Reward Possession” (dog’s desire to play with and hold a toy reward, including the force and determination to maintain grip on the reward), “Reward Persistence” (amount of time and effort dog spends attempting to recover an inaccessible reward), “Reward Focus” (dog’s ability to maintain visual focus on a reward despite distractions), and “Reward Arousal” (dog’s ability to regulate physical arousal [i.e., vocalizing, jumping, etc.] when presented with a reward placed just out of reach). Each measure was scored on a scale of 1–5 by an evaluator (CPS senior trainer), whose scoring on the assessment was validated and demonstrated high inter-rater reliability in previous studies (20, 21). The reward used in the behavioral test that these scores were derived from was the same as the reward used as the reinforcer in the current study (a ball). Scores were averaged to create an overall “Reward Value” score, and groups were balanced by this score as well as sex creating two groups of 14 (5 F/9 M per group), each with an average reward value score of 3.83 (SEM = 0.16 and 0.14 for marker and reward-only groups, respectively). One group was trained with the use of a clicker as a marker and signal for reward, and the other group was trained with reward only.

Dogs participated in the study in litter cohorts of 4–8 dogs as they entered the training phase at CPS. Each litter was evenly divided between the two groups, using the balancing system described above. Training was typically conducted daily (M-F), with the order of dogs varied to minimize potential order effects. Dogs wore a patrol harness (Blackthorn K9, Monroe, MI) that could be attached to a leash during all training and testing.

All training sessions occurred inside an empty room of the AUCVM with tiled flooring. Transparent barrier screens were used as walls to divide the training area where the dogs, handler, and trainer worked from the experimenter observation area. A GoPro video camera was mounted to the top of the wall and recorded all sessions. The experimenter sat behind the wall and observed and recorded trial information. At the end of each day, the floor of the training room was swept and vacuumed, and a door of the room leading to the outside was propped open for at least 15 min to air out the room.

The target odor used throughout the study was a 0.1% concentration of amyl acetate diluted in mineral oil (10^−3^ v/v). Amyl acetate was chosen as the target odor as it is commonly used in canine detection research due to its high volatility and handling safety (14, 22, 23). Each day prior to the start of training, 1.5 mL of the solution was pipetted onto a new cotton pad (Swisspers® 100% Cotton Rounds Pads), which was placed inside a jar for presentation during training.

### 2.3 Training

For both groups, correct responses were rewarded with a ball (Chuckit!® Ultra) and play with the handler. For dogs in the reward-only group, the trainer tossed the ball directly to the dog immediately upon performing the correct response. For dogs in the marker group, the trainer pressed a metal handheld clicker device (Gary Wilkes’ Mega-Click) producing a “click-clack” sound immediately upon the dog performing the correct response, followed by delivery of the ball. Dogs were allowed to continue playing with the ball and engage in interaction with the handler while the trainer set up the next trial. All dog activity was conducted by the same two individuals serving as the trainer and handler. The trainer, a professional detection dog trainer with significant experience using both methods for training detection dogs, delivered the marker signal and/or reward for all dogs. The handler, a professional detection dog trainer and handler, handled and managed all dogs during training and testing. The handler and trainer were aware of the target location throughout the training phase.

#### 2.3.1 Marker conditioning

For dogs in the marker group, the first session consisted of pairing the sound of the clicker with the delivery of the ball in order to create an association between the clicker and reward. This session consisted of 20 successive pairings of the clicker sound and the delivery of the ball. Following procedures used by Gilchrist et al. (11), the reward-only group received the same number of reward presentations in order to control for exposure to the reward and any associations created with the trainer or environment that could occur at this step for the marker group prior to beginning training. Instead of sounding the clicker, the trainer held and pressed a mock-clicker that did not make a sound to simulate the amount of time between marker and reward in the marker group (11) (only done at this step).

#### 2.3.2 Alert response training

Following the marker conditioning session all dogs were trained to sit, which was the operant response alert behavior that dogs would eventually be trained to perform to indicate detection of the target odor. The sit response is a common alert behavior used with detection dogs as it is considered a passive response (i.e., minimizes disturbance to the environment and risks to the dog or handler, compared to active responses such as barking or scratching at the target location) and is a clearly observable behavior that can be easily identified by the handler (24).

Alert response training was conducted in 20-trial sessions. The target behavior was sitting (hindquarters touching the ground) within 3 s of the verbal “sit” command, without any luring or repeating of the command. The behavior was initially shaped using a combination of luring (e.g., holding the ball over the dog’s head or taking a step toward the dog to encourage shifting back into a sit) and capturing if the dog spontaneously offered a sit, using the reward procedures corresponding to their group. The verbal “sit” command was added once the dog was reliably performing the sit, and physical cues were progressively faded out. Each time the dog was rewarded constituted one trial. Trials continued until the dog performed the correct target behavior on eight out of a moving block of 10 trials within a 20-trial session, at which point the dog advanced to the next phase.

#### 2.3.3 Odor detection pre-training

The next phase following alert response training consisted of a single 10-trial session to introduce dogs to odor box work, designed to teach the dogs to approach and investigate the odor presentation boxes and to begin to condition an association with the target odor. Purpose-built wooden odor presentation boxes (henceforth “tall boxes”) were used to facilitate the initial training process. The boxes were 72-cm tall at the highest point and had a slanted top with a nose hole designed to be at the height of the dog’s snout when standing or sitting, with a shelf inside the box designed to hold the jar containing the odor stimulus at the level of the nose hole. A hinged door on the back side of the box was designed to allow the trainer to insert the toy reward into the box and deliver to the dog while their snout was in the hole. To minimize cross-contamination due to the wooden material of the boxes used in this phase, boxes used to contain the target were kept consistent (i.e., boxes used to contain the target odor were never used as non-target boxes). Multiple designated target and non-target boxes were included in the larger set used throughout the study so that target and non-target boxes were routinely replaced and rotated throughout training.

Only one box, which contained the target odor, was present in this phase. On each trial, the handler walked the dog on leash to the front of the box while the trainer was positioned behind the box. On initial trials, the trainer held the ball in his hand, showed the dog the ball and then inserted the ball into the back side of the box and out through the hole on the front to entice the dog to the hole. As the dog approached, the trainer lured the dog’s snout into the hole in the box and then allowed the dog to take the ball. For the marker group, the trainer clicked while the dog’s snout was in the hole just before releasing the ball. The amount of luring was reduced across trials until the dog was independently approaching the box and investigating the hole without the reward visible. All dogs completed 10 trials before moving on to the next phase (i.e., there was no performance criteria to move on).

#### 2.3.4 Odor detection training

Next, dogs commenced odor detection training on a multi-box odor discrimination lineup. A training protocol consisting of five steps, adapted from Smith et al. (25) was designed to: (1) condition an association between the target odor and reward, (2) teach the dogs to search the boxes for the target odor, (3) perform the trained alert response to indicate the location of the target odor, (4) discriminate the target odor from non-target odors, and (5) perform the search and response sequence independently. At each step, there was a defined response requirement and criteria for moving to the next step. The response requirements and general complexity of the task (e.g., number of boxes, presence of non-target distractor odors) increased across steps, with training progressing from simple conditioning of recognition of the target odor to performing the final alert response consisting of sitting and orienting toward the target location. The requirements for a correct response at each step were defined by the latency, duration, and topography of the response (described below). The tall boxes used in the previous phase were used in the first two steps as the height and shape of the box were designed to facilitate performing the sit alert while encountering the target odor. The last two steps utilized shorter, flat boxes (30.5 × 30.5 × 19.5 cm) made of ultra-high-molecular-weight polyethylene with a hole in the top (henceforth “low boxes”). These boxes were wiped down at the end of each day with heavy duty disposable shop towels, and sprayed with high-pressure water inside and out and aired out to dry in the sun at the end of each week. As above, multiple designated target and non-target boxes were included in the larger set used throughout the study so that target and non-target boxes were routinely replaced and rotated throughout training. All sessions at each step of this phase consisted of a maximum of 10 trials, with each reinforcement event constituting a trial.

##### 2.3.4.1 Odor detection training step 1

In the first step, dogs were worked on leash and were trained to systematically sample a lineup of boxes. The goal behavior for this step was exhibiting a “change of behavior” (COB), a conditioned response to the target odor exhibited as a characteristic abrupt change in ongoing searching behavior indicative of target odor recognition (e.g., head turn, change in speed, direction, and/or sniffing pattern, freezing) (26), which was shaped using successive approximations and reinforced using the reward procedures corresponding to group. On each trial, the target odor was present in one of three tall boxes, with the other two remaining empty. For each dog’s first session at this step, the target box was placed in the first position of the lineup on Trial 1, moved to the second position on Trial 2, and third position on Trial 3 in order to encourage systematic sampling of the lineup. After the third trial of this first session, and on all trials on subsequent sessions of this step, the position of the target was pseudorandomized such that it did not remain in the same position for more than two consecutive trials. If the trainer felt that the randomized position could be counterproductive to the dog’s learning (e.g., the target was designated to be in the position where the dog had false alerted on the previous trial), the position could be changed at the trainer’s discretion, but this trial was automatically scored as incorrect. Dogs were required to exhibit a COB, holding their nose at the hole for 2 s, on a minimum of eight trials in order to advance to the next step.

##### 2.3.4.2 Odor detection training step 2

In this step, the number of tall boxes was increased to five, with one containing the target on each trial and the others remaining empty. Boxes were spaced approximately 60 cm apart. Dogs were worked on leash and were trained to perform the sit response at the target box using a combination of prompting, shaping, and luring. The criteria for the sit response at this step was the dog sitting with its head oriented toward the box within 10 s of sniffing the target box. Lines on the floor marked the distance dogs were required to be within when performing the response. At this step, the front paws were required to be within 60 cm of the target box when the dog sat. Dogs were required to perform the criteria-compliant response without any handler or trainer assistance (e.g., luring, body blocking, or verbal command), false alerts (performing the response at a non-target position) or misses (operationally defined as sniffing the target box and moving more than one position away) on a minimum of six trials, the last three of which were correct, in order to advance.

##### 2.3.4.3 Odor detection training step 3

At this step, the tall boxes were replaced with five low boxes and non-target materials were introduced as distractors and negative controls to require discrimination of the target odor from other odors. Non-target materials were always presented in jars identical to those used to hold the target, and at this step included mineral oil (the solvent used to dilute the target odor) on a cotton round and a nitrile glove (worn by experimenters when preparing and handling stimuli), with one box containing an empty jar and the remaining left empty. In the first session of this step, on trials 1–6 the target box was placed in position 1, 1, 2, 3, 1, and 5, respectively, in order to facilitate transition to the new boxes and encourage systematic searching past non-targets. After the sixth trial of this first session, and on all trials on subsequent sessions of this step, the position of the target was pseudorandomized as above. Dogs were worked on leash and the response requirement consisted of sitting with all four paws within 60 cm of the target box within 3 s of sniffing the target box, and head orienting toward the target box within an additional 3 s of sitting, trained using a combination of prompting, shaping, and luring. Dogs were required to complete a minimum of six trials in the session, and to perform the response requirements with no assistance and no false alerts or misses (as defined above) for the last four trials consecutively in order to advance. If dogs did not meet this criteria within 10 sessions, they automatically advanced to Step 4.

##### 2.3.4.4 Odor detection training step 4

In this final training step, the response requirements were the same as Step 3 except that the dog was required to search the lineup off leash, perform the entire response (sit and orient) within 3 s of sniffing the target box, and hold the response for a minimum of 2 s. The handler was required to remain behind the start of the lineup during all trials, and an area to the side of the lineup was designated where the trainer was required to remain during the trial (approximately 2.5 m away from the lineup), in order for a trial to be considered correct. These conditions were added to increase the independence required of the dog while searching and minimize any potential influence from the non-blind handler and trainer. In order to complete this step and advance from the training phase, dogs were required to either complete a minimum of six trials with the last four performed correctly, or complete a maximum of 10 sessions at this step, whichever came first.

All training sessions were live-scored by the experimenter who recorded dogs’ performance on each trial in terms of a target odor indication (i.e., hit or miss), whether a false alert occurred, whether the response criteria for each component was met according to the criteria for that step, and whether the dog met the criteria to advance to the next step. Total trials in each phase/step was calculated for each dog. Dogs that automatically advanced from a phase after reaching the maximum number of sessions were assigned an additional six trials, which was the minimum number of trials in which they could have met criteria had they not been automatically advanced.

### 2.4 Post-training assessment

After completion of training, a 10-trial post-training assessment was conducted. The session was run in the same way and with the same scoring criteria as the final training step, except that the handler was blind to the location of the target on each trial. Additionally, the non-target distractors used in training were replaced with novel distractors (with the exception of mineral oil which remained): permanent marker ink, paprika, and fish oil capsules (all presented via cotton rounds odorized with the distractor substance). As before, the trainer clicked and/or rewarded the dogs, and the experimenter live-scored each trial for target odor indications (coded as a “hit” or a “miss” as described above for the training phase) and false alerts, with components of the alert response scored using the same criteria as Step 4 of training. A total response score (sum of scores for each component, with a total of four possible) was also calculated for each trial. For response latency and distance, rather than the binary variable of whether or not the response component criteria were met, actual latency to respond (measured in number seconds, rounded to the nearest second) and distance were scored from video to allow for a more meaningful measure of these aspects of performance. Distance from the target box was measured by pausing the video when the dog sat and counting the number of floor tiles (15 cm x 15 cm) between the outer edge of the box and the dog’s front paws.

### 2.5 Context generalization

Following the post-training assessment, dogs were tested for generalization of the trained behavior in an operational search task. Six targets were hidden throughout six distinct areas inside an athletics complex on the Auburn University campus (e.g., dressing rooms, locker rooms, storage areas, and offices), and were placed inside cabinets, drawers, or boxes to visually conceal the target and add a layer of depth to the hide. Two distractors (a glove and a cotton round with mineral oil) were also placed throughout the search area. Dogs performed one continuous search for the six targets and were rewarded according to their group by the trainer as in previous phases. The experimenter recorded searches with a handheld camera. The first five targets were searched on leash, with the last conducted off leash. Searches were treated as a hybrid training/testing session with the handler and trainer aware of the target locations. That is, the dog led the search in front of the handler and was given the opportunity to independently perform the response once they encountered the odor and showed a COB indicative of odor recognition. If the dog successfully located the odor and demonstrated a COB but did not offer the alert response, the trainer, who remained at a distance behind the handler while the dog searched, intervened by giving the verbal “sit” command. If the dog searched the area where the target was located and did not show a COB, the search continued and that target was scored as a miss. The trainer clicked and/or rewarded the dogs as before.

Each search was scored live by the experimenter for hit or miss and false alerts. Hits were scored as either unassisted (dog performed alert response with no trainer intervention) or assisted (dog showed a COB but required trainer intervention to perform the alert response). Misses were recorded if a dog searched the area where a target was located and did not alert or show a COB. Each unassisted alert was also scored for (1) dogs’ distance from the target when responding, either as “at source” (dog was within approximately 1 m of the target) or “not at source” [dog demonstrated COB to target odor and responded but was more than 1 m from the target, also known as a fringe response (27)], and (2) precision of dogs’ alert, as either correctly indicating the location of the odor source (head/snout pointed in the direction toward the location where the target was hidden) or away from source (head/snout pointed in a direction away from where the target was located) as binary variables from video.

### 2.6 Resistance to extinction

The final session was based on methods by Smith and Davis (12) and consisted of a 10-trial session similar to the post-training assessment except that the primary reward for correct responses was withheld for all dogs. For dogs in the reward-only group, alerts to the target were ignored and the handler waited until the dog stood up and moved to the next box, at which point the handler called the dog away from the boxes in a neutral tone. If the dog did not leave the target after 10 s of performing the response, the handler recalled the dog in a neutral tone and/or gently guided the dog away from the boxes using the handle on their harness. For dogs in the marker group, responses to the target odor were marked with the clicker followed by the handler recalling the dog in a neutral tone and/or gently guiding the dog away from the boxes using the handle on their harness. For both groups, the trial ended if the dog missed a target (i.e., sniffed the box and moved more than one position away without responding). On all trials, the handler was instructed not to praise the dog and simply re-set the dog at the start position for the next trial. Trials were scored the same way as the post-training assessment.

### 2.7 Data analysis

Inter-rater reliability (IRR) was assessed by independent scoring of a random subset of sessions for each dependent measure. For odor discrimination task variables, a research assistant that was unaware of the study aims and hypotheses scored 15% of the sessions from video. IRR between the two raters was good (latency: Kendall’s *W* = 0.70, *p* = 0.003; distance: *W* = 0.79, *p* < 0.001; orientation: kappa = 0.83, *p* < 0.001; duration: kappa = 0.62; *p* <. 0.001; hit: kappa = 0.89, *p* < 0.001). For searches, a professional detection canine trainer that was unaware of the study aims and hypotheses (as well as the experimental groups as the reward procedures were out of frame and without sound) scored 15% of the searches. IRR was good for both variables (distance: kappa = 0.87, *p* < 0.001; precision of indication: kappa = 0.73, *p* = 0.003).

Differences in acquisition of the task were analyzed with Generalized Linear Models (GLM) with group as a fixed factor and total trials to criteria in each step of the training phase as dependent measures. Dogs’ average “Reward Value” score from the pre-study behavioral assessment was included as a covariate, along with its interaction with group, to account for variability in individual differences in motivational traits that could influence learning and the effectiveness of a training procedure (25). A Gaussian distribution was used for all analyses unless otherwise specified.

Differences in odor detection accuracy measures and alert response topography during the post-training assessment were analyzed using Generalized Linear Mixed Models (GLMM) with group and trial number as fixed factors and dog ID as a random factor. A binomial distribution was used for binary variables (hit/miss, false alert, and whether the orientation and duration criteria were met on each trial).

Context generalization from the odor discrimination task to the operational search was assessed with a GLM with group as a fixed factor and total number of unassisted alerts as the dependent measure. For response metrics, because only searches in which unassisted alerts occurred could be analyzed and the number of unassisted alerts varied across dogs, the dependent variables of interest (distance and precision) were calculated as a proportion of the number of unassisted alerts each dog had, with number of alerts as a covariate and group as fixed factor.

Group differences in odor detection and alert response measures during the extinction test were analyzed using the same models as the post-training assessment analyses. For analyses of alert response metrics, only trials in which dogs alerted to the target were included (i.e., trials with an absence of a response were omitted rather than scored as a failure to perform each aspect of the alert response). The interaction between trial number and group was assessed to examine cumulative effects of extinction across the session. If the interaction was significant, a separate model was conducted for each group.

Finally, to examine whether potential group effects were due to differences in delays between response and reinforcement (11), a subset of videos were coded for the delay (in seconds) between a correct response and the sound of the click (marker group only) and between a correct response and the primary reward (all dogs). A subset (15%) of dogs’ last session in the Odor Detection Training Step 3 (half from the marker group and half from the reward-only group) were randomly selected. This step was chosen as it was the last training step in which the trainer’s position was not restricted, which could influence the delay and potential effects. Additionally, a random subset (15%) of sessions from the post-training assessment (half from the marker group and half from the reward-only group), in which the trainer was required to remain at a distance, were coded. Differences in latency to primary reinforcement were analyzed using Generalized Linear Mixed Models (GLMM) with group as a fixed factor, dog ID as a random factor, and latency to reinforcement in seconds as the dependent variable.

## 3 Results

### 3.1 Odor detection training

Four dogs (three in the reward-only group and one in the marker group) failed to meet the Step 3 criteria within the maximum number of sessions allowed and automatically advanced to Step 4. Of those dogs, all but one (reward-only group) subsequently met criteria in Step 4. This same dog and an additional four dogs, all in the reward-only group, reached the maximum number of sessions in Step 4 without meeting criteria. Table 1 shows mean number of trials to complete each training phase for both groups. Dogs in the marker group required significantly fewer trials to complete the final stage of odor detection training (Step 4) than the reward-only group (*z* = −2.65, *p* = 0.008). There were no differences between groups in number of trials to meet criteria in the other phases (*p*s > 0.34). There were no interactions between “Reward Value” scores and group; however, there was a main effect of “Reward Value” on number of trials to complete the Alert Response Training phase, where higher scores (i.e., higher levels of reward motivation) were predictive of a greater number of trials required to meet criteria (*z* = 2.55, *p* = 0.011). On average, the clicker was sounded 1 s after a correct response was performed by dogs in the marker group. The time (in seconds) between a correct response and primary reinforcement (ball) did not significantly differ between the marker (*M* = 3.13, SE = 0.55) and the reward-only groups (*M* = 3.54, SE = 0.22), *p* = 0.46.

**Table 1:** Average number of trials (+/− standard error of the mean) in each training phase by group.

Group	Alert response training	Odor detection training - Step 1	Odor detection training - Step 2	Odor detection training - Step 3	Odor detection training - Step 4
Marker	51.9 (± 5.32)	39.2 (± 4.38)	40 (± 6.24)	44.8 (± 6.13)	**36 (± 7.10)**
Reward only	60.1 (± 7.68)	34.7 (± 4.24)	43.9 (± 8.76)	50.4 (± 9.30)	**68.2 (± 9.91)**

Bolded text indicates a significant difference between groups.

### 3.2 Post-training assessment

There was no significant difference in probability of a correct detection (hit) between the marker group (*M* = 93.57%, SE = 1.69) and the reward-only group (*M* = 97.86%, SE = 1.14) in the post-training assessment (*p* = 0.09). Probability of a false alert also did not differ between the marker and reward-only groups (*p* = 0.98, *M* = 7.14%, SE = 2.66, and *M* = 7.14%, SE = 2.44, respectively).

**Figure 1:**
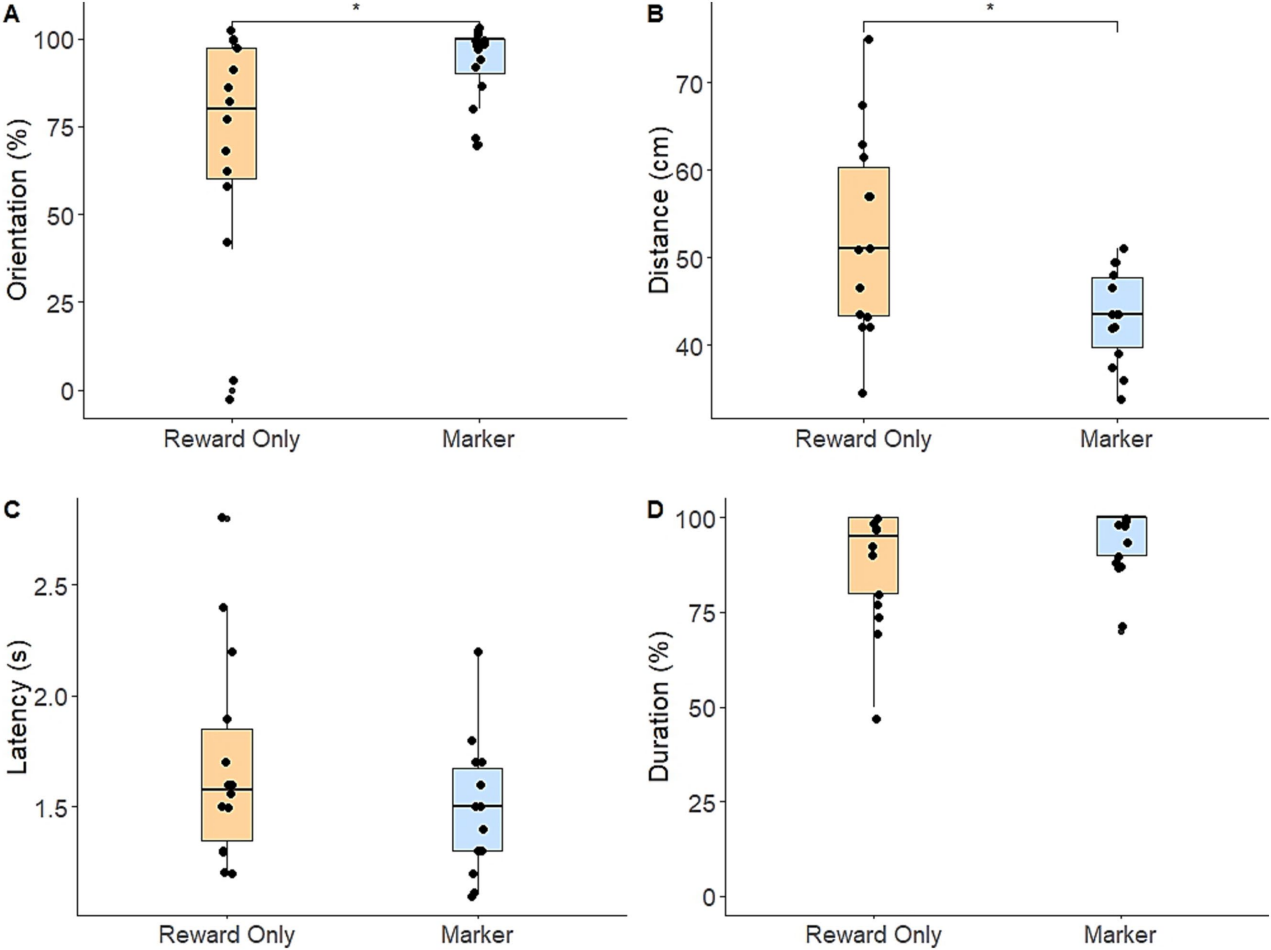
Box-and-whiskers plots showing dogs’ performance of each component of the alert response in the post-training assessment [**(A)** percentage of trials in which dogs correctly oriented; **(B)** dogs’ distance from the target box; **(C)** dogs’ latency to perform the alert response; **(D)** percentage of trials in which dogs met the response duration criteria]. Horizontal bars indicate median (blue: marker group, orange: reward-only group) and dots represent individual dogs. Brackets and asterisks show statistically significant differences.

Regarding the alert response, dogs in the marker group had a significantly higher total response score compared to the reward-only group (*t*(26) = 3.481, *p* = 0.002). Specifically, dogs in the marker group were significantly more likely to orient toward the target box (*M* = 92.1%, SE = 2.79) than dogs in the reward-only group (*M* = 69.3%, SE = 8.59; *z* = 2.33, *p* = 0.02; Figure 1A), and responded significantly closer to the target box (*M* = 43.2 cm, SE = 1.43) than dogs in the reward-only group (*M* = 52.5 cm, SE = 3.07; *t*(26) = −2.728, *p* = 0.011; Figure 1B). There were no significant differences in latency in seconds to respond (marker group: *M* = 1.49, SE = 0.07; reward-only group: *M* = 1.70, SE = 0.19) or likelihood of meeting the response duration criteria (marker group: *M* = 94.3%, SE = 2.12; reward-only group *M* = 87.9%, SE = 3.93; *p*s > 0.19) (Figures 1C,D).

On average, the clicker was sounded 1 s after a correct response was performed by dogs in the marker group. The time (in seconds) between a correct response and primary reinforcement (ball) did not significantly differ between the marker (*M* = 3.61, SE = 0.36) and the reward-only groups (*M* = 3.65, SE = 0.32), *p* = 0.46.

### 3.3 Context generalization

One dog in the marker group appeared to be distracted by the presence of excessive environmental disruptions during the search test (e.g., large crowds of people and noise due to a cheerleading camp in the building) resulting in not actively searching during the test, therefore this dog was excluded from the search test. Eight dogs from each group independently performed the alert response upon their first target odor encounter of the search. Of those that did not perform the trained alert (sit) to the first target, four of the six in the reward-only group and all five of the dogs in the marker group demonstrated odor recognition (i.e., COB but no trained alert). There was no difference between groups in the average number of independent alerts across all six targets (marker group: *M* = 3.92, SE = 0.57; reward-only group: *M* = 4.14, SE = 0.51; *z* = − 0.28, *p* = 0.78). There were no false alerts in response to the planted distractors or other non-target items. Dogs in the marker group on average had a greater proportion of alerts that indicated toward the target (59.2%, *SE* = 9.32) than the reward-only group (37.9%, SE = 9.91), *z* = 1.97, *p* = 0.04, with no difference in response distance (marker group: 82.63%, SE = 8.11; reward-only group: 83.94%, SE = 5.42), *p* = 0.88.

### 3.4 Resistance to extinction

**Figure 2:**
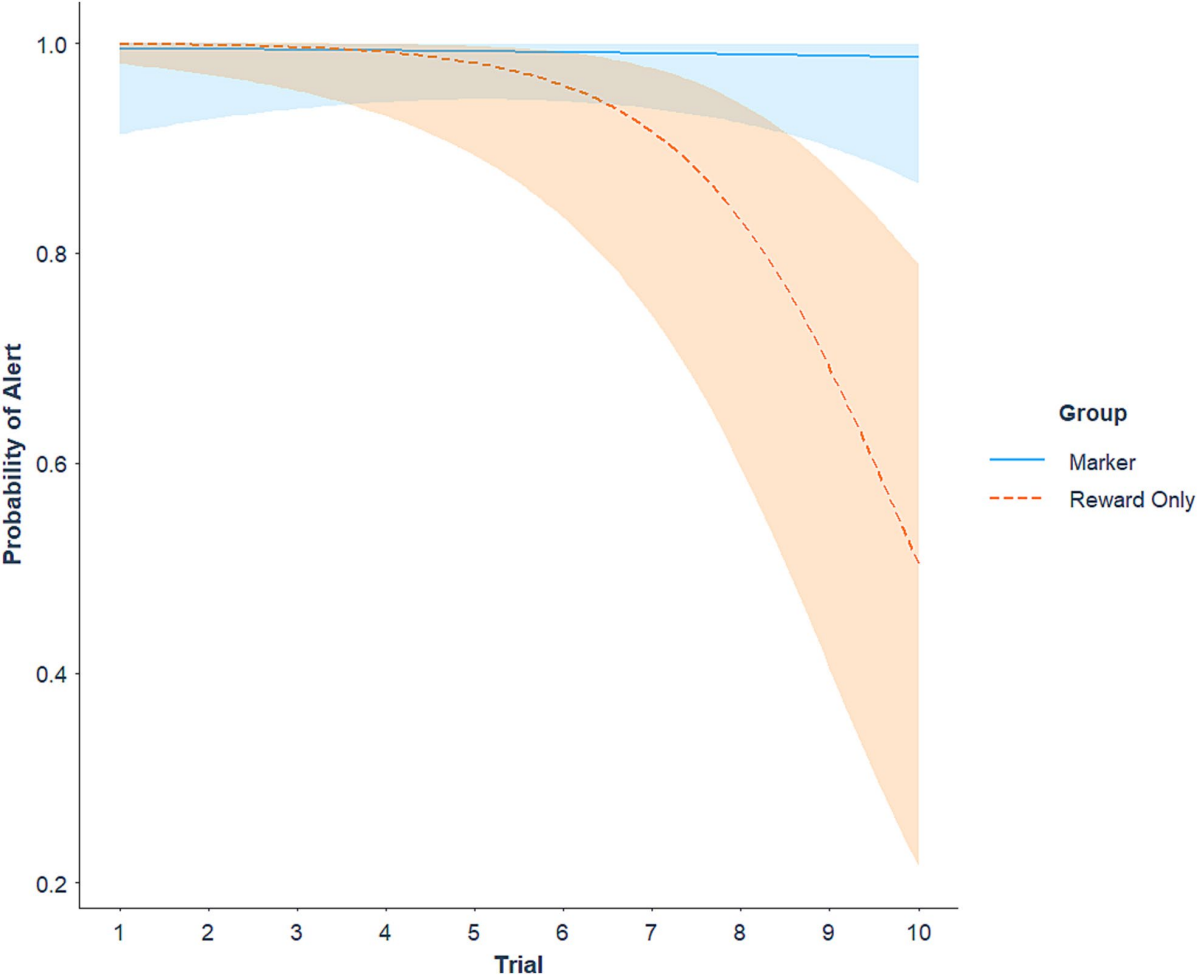
Probability of an odor detection alert (hit) across trials in the extinction session, represented as a predicted logistic trajectory. Bands represent 95% confidence intervals.

Average sensitivity (number of hits divided by number of trials) in the extinction session was 97.85% (SE = 1.14) for the marker group and 85.47% (SE = 3.96) for the reward-only group. There was an interaction between group and trial number for probability of a hit (*z* = 2.35, *p* = 0.019), where the likelihood of alerting to the target odor decreased across non-rewarded trials for the reward-only group (*z* = −3.90, *p* < 0.001), with no change across the session for the marker group (Figure 2). Average false alert rate (FAR; number of false alerts divided by number of trials) in the extinction session was 2.86% (SE = 1.63) for the marker group and 0.71% (SE = 0.71) for the reward-only group. There was a main effect of trial in which the probability of a false alert overall decreased across extinction trials (*z* = −2.03, *p* = 0.042), but did not differ between groups (*p* = 0.314).

Analysis of aspects of the alert response on trials in which alerts occurred revealed that the marker group had a higher response score across the extinction session than the reward-only group (*t*(25) = 4.76, *p* < 0.001). Main effects of both group and trial were observed for response duration and distance, where likelihood of meeting the response duration criteria (*z* = −2.18, *p* = 0.029) and distance to the target box (*t*(225) = −2.15, *p* = 0.033) decreased across trials, but overall the marker group was more likely to meet the duration criteria (*z* = −2.18, *p* = 0.146) and responded significantly closer to the target box (*t*(26) = −3.80, *p* < 0.001) than dogs in the reward-only group. Dogs in the marker group were also more likely to orient toward the target box than the reward-only group (*z* = 2.19, *p* = 0.029), with no effect of trial (*p* = 0.87). For latency to respond, there was an interaction between group and trial (*t*(226) = −2.411, *p* = 0.017) in which the latency to sit significantly increased across trials for dogs in the reward-only group (*t*(105) = 2.31, *p* = 0.023), with no change for dogs in the marker group (*t*(121) = −0.829, *p* = 0.41).

## 4 Discussion

The current study examined the effectiveness of training detection dogs with the use of a marker as a signal for reward. Dogs in the marker training group were trained with a clicker to mark correct responses, followed by delivery of an established reward (a ball). Dogs in the reward-only group were rewarded with the ball upon a correct response. Benefits of marker training were observed in multiple aspects of performance, resulting in reduced training time and more optimal behavior, summarized in Table 2 and discussed in depth below.

**Table 2:** Summary of general effects of marker training across each phase of the study.

Domain	Method	Measure	Advantage of marker?
Task acquisition	Training	Number of trials to complete training	Yes
Task proficiency	Post-training odor recognition test	Sensitivity	No
		Specificity	No
		Topography of response	Yes
Context generalization (transfer of learning)	Search test in novel operational setting	Number of unassisted alerts	No
		Topography of response	Yes
Resistance to extinction	Odor recognition test with no primary reinforcement	Sensitivity	Yes
		Specificity	No
		Topography of response	Yes

“Advantage of marker” indicates that dogs trained with a marker significantly outperformed dogs in the reward-only group for at least one aspect of the corresponding variable.

Dogs trained with a marker required fewer trials to complete the training phase compared to dogs trained with the reward alone. Differences in speed of learning were apparent in later stages of training when the task and response criteria became more complex (i.e., dogs were required to perform the alert response at increasingly closer distances to the target odor, for longer durations, with a more precise posture, and at a greater distance from the trainer and handler). Additionally, marker-trained dogs performed the alert response with greater physical precision and proximity to the odor source in the post-training assessment compared to the reward-only group, suggesting that differences in training time observed between groups were in part due to these components of training. One possibility is that the performance of the two groups diverged when the difficulty increased in the final step due to advantages and limitations of using or not using a marker, as marker training has been suggested to be more effective for training more complex behaviors (28). However, it is likely that the reward procedures began to influence behavior at earlier stages, but the effects were not captured due to looser criteria for acceptable responses in those stages (e.g., longer acceptable latencies to respond, greater distance from the target). It should be noted that some dogs were advanced after reaching a maximum allotment of training trials rather than when they met the training criteria, which could have influenced the observed results compared to actual training time if they had been allowed to continue. However, all dogs that automatically advanced from training were in the reward-only group, therefore additional training would have only further enhanced the observed difference in training time between groups. The inclusion of dogs in the testing phases that were advanced without meeting training criteria also likely contributed to the observed differences in performance and it is possible that, had all dogs been allowed the opportunity to continue training until meeting criteria, the differences would have been minimized. However, the current results demonstrate that dogs trained with a marker for a given amount of time outperformed dogs trained without a marker that were allotted the same amount of time.

Differences in response topography between groups likely reflect the precision required of the operant response, which could easily be marked with the clicker the moment the behavior occurred but was more difficult to time using the primary reward. For example, capturing the desired orientation and distance for dogs in the marker group only required the trainer to click as soon as the dog performed the correct behavior. For dogs in the reward-only group, capturing the correct response required skilled timing and accuracy of throwing the ball so that it landed in front of the dog at the right moment. The inherent delay between the dog performing the correct response and receiving the reward also created the opportunity for the dog to move out of the correct position, potentially resulting in reinforcement of incorrect behavior. As a consequence, dogs typically learned to look up in anticipation of the reward, look back at the trainer, or other superstitious behaviors (for example, one dog developed a paw-lift behavior when responding) which occur when animals learn that a behavior that was unintentionally reinforced is associated with reward (29). This often required the trainer to position himself at a closer distance to be able to reward the desired behavior, which was incompatible with the final level criteria that the trainer and handler remain at a distance from the dog. Similar patterns were observed for other components of the response such as the dog’s distance to the target; for example, in anticipation of catching the ball, dogs often began backing up which resulted in reinforcing responding at a further distance than intended. These findings are in line with the notion that effects of marker training may be more evident when training complex and nuanced behaviors requiring precise timing or greater distance from the trainer (28). However, it should be noted that the effect of marker training is not simply related to the physical form of an operant alert response; the ability to minimize the delay in between contact with odor and reward is critical for conditioning a strong association with the target odor that will guide subsequent behavior (3). Coding of the amount of time that passed between the dog performing a correct response and receiving reinforcement supports the function of the marker in reducing the delay, as the delay between a response and the mark was only 1 s, whereas dogs in the reward-only group experienced a delay of over 3 s between a correct response and reinforcement, similar to the delay between response and primary reinforcement for the marker group.

The specific response requirements of an alert behavior may vary across organizations and be less stringent than required in this study, in which case the advantage of using a marker may be less apparent. For example, the “focus response” used here, in which the dog was required to orient toward and stare at the target box, may not be critical for a dog to effectively communicate a find. However, depending on the search environment, precision in identifying the location of the target may be important (30). Regardless of the importance of the dog’s precision in orientating toward the target location, the use of a marker can minimize other undesirable behaviors such as looking at the person delivering the reward which can result in handler dependence and unintentional cueing. The use of a marker also minimizes these behaviors by allowing the dog to work at a distance from the handler and/or trainer. In terms of the dog’s distance from the target odor, while the specific distance required in this study was arbitrary and may have inflated differences between groups compared to if the acceptable distance had been greater, being able to pinpoint the source of the target (as opposed to a “fringe” response in which the dog responds at a distance) is important in many operational scenarios, with responses at even closer distances preferred (30). Further, while responding in close proximity to the source of the odor is desirable, concerns may arise when the dog is rewarded near the odor using traditional reward delivery methods (i.e., throwing a ball for the dog to chase). For example, the ball or the dog can come in contact with the target, resulting in contamination of the odor. In the case of volatile explosives, the physical impact of the ball landing on the target, or the dog running into the target when chasing the ball, can trigger a detonation. For this reason, training with certain volatile explosives that are highly shock and friction sensitive often involves limitations on access and exposure to the explosive material (31), for example requiring that the dog be rewarded “away from source,” which raises concerns over reinforcing undesirable behavior or weakening the odor-response association. Our results indicate that these challenges can be addressed using marker training.

Other aspects of detection performance in the post-training assessment did not appear to be affected by using a marker. For example, while marker training resulted in an alert response that more precisely indicated the odor source in terms of distance from and orientation toward the target location, there was no effect on latency to perform the alert (i.e., amount of time between recognition of odor and performing the operant response) or the dogs’ ability to hold the response for the required 2-s duration. Dogs tended to respond immediately after detecting the target odor, suggesting that once learned, the detection-response behavior chain is rapid with little room for improvement from the use of a clicker. For duration of the response, the criteria may have been too short to demonstrate any group effects, which may have occurred with longer and more challenging response duration requirements. Similarly, there were no group differences in sensitivity or specificity of target odor alerts, which were overall high for both groups. The lack of effects for these variables could be due to the nature of the behaviors and their response requirements, and potential ceiling effects resulting from the training phase. That is, dogs were required to reach a high level of proficiency on multiple aspects of performance with stringent criteria, or complete an extensive amount of training, to advance to the post-training assessment. As such, the odor-response association was likely well-learned by the time the test occurred, potentially washing effects for less nuanced behaviors. For example, sensitivity was calculated based on hits, which were recorded when a dog alerted to the target odor regardless of whether the criteria for all components of the response were satisfied. Similarly, false alerts to non-targets rarely occurred, likely due to the thorough discrimination training and exposure to different types of distractors prior to testing. Had odor detection accuracy been assessed at earlier stages of training, or less stringent training criteria used, it is possible group differences in these variables may have been detected. These differential effects on different aspects of performance further suggest that the efficacy of marker training is most apparent for more nuanced and complex behaviors that, even after being trained, are more vulnerable to variability and obstacles in timing and precision common to traditional methods of reinforcement (28).

Following training and testing in the odor discrimination setting, we examined whether dogs could generalize what was learned in that setting to a free search setting in which dogs had never encountered or alerted to a trained odor, and whether marker training facilitates or hinders generalization. Results of the context generalization test demonstrated a moderate level of generalization for both groups, with half of the dogs from each group independently locating the target odor and performing the trained alert upon their first encounter of it in a search setting, and dogs in both groups performing the independent alert response to the majority of the targets across all six searches. This suggests that marker training of the alert response in the more artificial odor discrimination training environment did not result in context-specific behavior that impeded performance in a more natural search environment compared to training without a marker. Moreover, contrary to one study that found no effect of marker training on generalization of a trained behavior (9), marker-trained dogs demonstrated a better ability to communicate the exact location of the target in a novel and markedly more complex search environment, which has practical value for operational performance in which timely identification of the odor source is critical (30). This effect could be considered operationally more important than the distance of the dog to the odor source for which no difference was found, which could have been due to logistical constraints in the sizes of the search areas used.

Dogs were also tested under extinction to evaluate the effects of conditioned reinforcement on extinction of the trained response. The ball reward was withheld for both groups, but the marker group continued to receive a click following a correct response. Target odor misses (i.e., dog sniffed the target box but did not alert) increased across the session for the reward-only group, indicating extinction of the odor alert response. By contrast, alerts to the target odor remained high across the entire session for the marker group. False alerts decreased across the extinction session for both groups. Taken together, these results suggest that extinction increased sensitivity and specificity for the marker group (i.e., higher responding to the target odor and lower responding to non-target odors), and decreased responding to odors altogether for the reward-only group. When dogs in the reward-only group did alert to the target odor during the extinction session, effects were also observed on the topography of the alert response, with components of the response either unaffected or decaying to a lesser degree for the marker group compared to the reward-only group.

The increased resistance to extinction for the marker group suggests that the marker itself had acquired reinforcing properties, which is consistent with the ‘Reinforcing Hypothesis’ that predictor signals can function as a reinforcer [(6, 12); but see (7) for other explanations]. This conditioned reinforcer effect is supported by evidence that a reward signal produces a release of dopamine similar to levels that occur during actual reward receipt (6). Our results contrast findings by Kalafut et al. (32) demonstrating disruption of a trained response when not every click was followed by a reinforcer, but this difference could be due to the nature of the task (e.g., performance of a behavior chain in a free operant task versus the discrete trials procedure of the current study). Excessive use of a marker in the absence of reward should be avoided as it can produce negative affective states (33) and will eventually result in extinction of the mark as a conditioned reinforcer (12). However, the results of the current study suggest that conditioned reinforcers can occasionally be effective in maintaining behavior when providing primary reinforcement is not possible or feasible. For example, it may be impractical or hazardous to reward dogs in certain conditions. In such cases, handlers could mark the behavior to provide feedback regarding a correct response in order to avoid extinction. This could be especially valuable given that animals easily learn contextual differences associated with reinforcement, with studies showing that detection of targets by trained dogs and rats declined in contexts they had learned to associate with the absence of reward (34–36). It should be noted that a true extinction protocol was not implemented for the marker group, which would require withholding of the conditioned reinforcer as well. The purpose of still delivering the mark for correct responses was to test its reinforcing properties and whether marked responses were more resistant to extinction, which has practical applications. Whether behavior that was trained with the use of a conditioned reinforcer is more resistant to extinction when all forms of reinforcement are withheld should be further examined, as well as the effectiveness of other types of reinforcers (for example, a lower value reinforcer such as verbal praise) in maintaining behavior.

There are some aspects of marker training that warrant further examination. For one, the number of pairings needed between the neutral signal and the reward during the initial conditioning of the marker to establish it as a conditioned reinforcer is unclear. The current study paired the sound of the click with the ball 20 times before beginning training, which was the number of pairings used in prior studies (10, 11). However, whether these many pairings are necessary or potentially counterproductive is unknown. For example, while the intention of the pairing trials is to simply present the neutral signal with a reward to establish an association, there is the potential for behaviors that occur during the reward presentation to be reinforced. In the current study, the trainer attempted to avoid reinforcing behaviors such as the dog looking at the trainer, which dogs began to do in anticipation of the reward, and attempted to avoid reinforcing the repetition of particular behaviors to minimize the development of superstitious behavior. Some have suggested that dogs learn the marker-reward association in as few as one to three presentations (9, 37, 38), while some do not pair the marker with the reinforcer prior to beginning training (28, 39). The latter practice may be most efficient if dogs are able to learn the meaning of the marker while simultaneously learning the task. The number of pairings needed to establish a marker as an effective conditioned reinforcer depends on factors related to the marker and the reward, such as stimulus intensity and magnitude. For example, pairing a marker with a highly preferred reward should lead to more rapid and stronger conditioning (40), which also likely depends on motivational characteristics of the dog. In a population of dogs bred for strong motivation and reward engagement (41), few, if any, pairings are likely needed to condition an association with the marker prior to beginning training and may be more efficient. Future research should examine these factors and the optimal methods for establishing a marker as a conditioned reinforcer.

Trainers typically fade the use of the marker once the learned behavior is well-established (7). While the current study did not include a formal examination of effects of fading the marker, two clicker-trained dogs were tested in a pilot session without the use of the clicker and demonstrated no disruption of performance. While anecdotal, this observation as well as the authors’ experience training a large volume of detection dogs using a clicker during initial learning stages and then transitioning to either a verbal marker or fading the use of a marker altogether, suggests that dogs becoming reliant on the marker to perform the task is unlikely (42). While the current study demonstrates the benefits of using a marker for the initial training of inexperienced dogs learning a new task, marker training can also be effective in improving previously trained behaviors or correcting undesirable behaviors (5). Future studies should examine the effectiveness of implementing marker training using a within-subjects design, comparing performance on a task originally trained without a marker to performance following the adoption of a marker.

A potential limitation that should be noted when considering the results of this study is that the same professional trainer was used throughout the study who was experienced both in detection dog training in general as well as in marker training. While this can be considered a strength in terms of consistency across dogs, future studies should examine whether similar effects are found across individuals, and whether level of experience regarding marker training is a factor (11). The fact that differences were seen with a trainer with years of experience both with and without marker training suggests that marker training may be even more beneficial for more inexperienced trainers that are less practiced in reward delivery timing and in being cognizant of and controlling for unintentional behaviors. However, timing of the marker is also critical when training precise behaviors, and poor timing is a frequent error in marker training that can lead to rapid acquisition of undesirable behaviors (43). Whether errors in marking are less forgiving than errors in primary reinforcement delivery is unknown and is worthy of further examination.

Limitations on blinding of experimental personnel should also be noted. Single-blind (handler is unaware of target location) or double-blind (all observers are unaware) procedures are optimal in assessments of detection dog performance (44). However, it is customary for handlers and trainers to not be blind during training stages in order to anticipate and respond appropriately to the dog’s behavior in a timely manner during critical learning opportunities (14, 25, 45). Furthermore, delays imposed by blind procedures requiring relaying communication of the dog’s response and outcome would complicate accurate assessment of the training methods in relation to reinforcement timing. In the final stage of training, dogs were required to work off leash with the handler and trainer remaining in a designated position at a distance from the dog, minimizing any potential influence on the dog’s behavior. The finding that differences in learning between groups were only observed in this final stage where handler and trainer involvement was limited, and not in earlier stages where interaction was greater, suggests that any impact of possible cueing on observed effects between groups was unlikely. Further support is demonstrated by the high performance by both groups in the post-training assessment when the handler was required to be blind to the target position. In terms of scoring of the dogs’ performance, as in similar studies, it was not possible to conceal the dog groups or conditions due to the real-time feedback of their reward procedure. However, potential observer bias in scoring was minimized by the use of objective, operational definitions for the behaviors scored, and was further addressed by checking inter-rater reliability with scorers that were blind to the study purpose and hypotheses (11). The context generalization searches were also run non-blind as this was the dogs’ first time encountering such a scenario and was therefore designed to be treated as a hybrid testing/training session, allowing the trainer or handler to intervene if needed (which was taken into consideration in the scoring). Standardization of important factors such as the individual handling the dogs and the placement of the hides also prevented searches from being blind. Potential influence from the handler or trainer was minimized as much as possible by requiring that they remain behind the dog, allowing the dog to lead the search independently. As above, potential bias in the scoring of the searches was addressed by checking inter-rater reliability with a scorer that was blind to the study purpose and hypotheses. Challenges in implementing blinding procedures for the evaluation of training methods could be addressed in future studies with technology to automate determination of dogs’ responses, scoring, and reward delivery (46–48), though the trade-off between technology-augmented experimental control and important nuance in the craft and skill of training should be considered.

The use of a sample of dogs from the same population confers advantages in minimizing variability due to subject characteristics, but may limit the generalizability of the findings. Although the effectiveness of markers as conditioned reinforcers is expected to be applicable across a range of populations, future research should aim to extend the results to include dogs of different breeds, ages, and backgrounds. Similarly, there are likely to be individual differences in the effectiveness of certain training methods, with some methods being more or less effective for different dogs. For example, the variability in performance within the reward-only group, with a select few dogs performing comparably to the marker group, illustrates that optimal performance is achievable without marker training in some cases. However, the lower variability and overall high performance in the marker group suggests that there were no dogs for which marker training was ineffective. While our results demonstrated that the effects observed between groups did not depend on individual differences in reward motivation, we did find an overall effect where higher reward motivation predicted slower acquisition of learning to sit in the first training phase. This seemingly counterintuitive effect could be due to high levels of reward motivation increasing arousal and interfering with learning (49). However, this effect was not seen in the detection training phase. A recent study from our group with a different population of dogs found that a measure of reward motivation was predictive of faster acquisition of an odor detection task (25), suggesting that effects of motivation on learning could vary depending on the nature of the task. Future research should examine how individual differences in cognitive and behavioral traits may influence the effectiveness of various training methods for different types of tasks, so that methods can be tailored to an individual dog’s needs. Finally, dogs are not the only species trained for odor detection work; for example, rats trained to detect landmines (34), people (50), diseases (17), and illegally trafficked wildlife (51) offer unique advantages for particular detection applications. Future research should examine whether these effects apply to other species differing in size, anatomy, or other species-specific factors that may influence optimal training methods.

## 5 Conclusion

This study is the first to examine the effectiveness of marker training for the application of training detection dogs, and provides the first empirical evidence of marker training improving task acquisition in dogs. Overall, results of this study indicate that marker training can increase efficiency and effectiveness of detection dog training, especially when there is an unavoidable delay in rewarding the dog, the dog is working at a distance, or the dog cannot be rewarded at source. These advantages of marker training are likely due to reducing the delay between odor recognition/response and reinforcement, thereby minimizing dogs’ confusion that often results in a weaker association with odor and the development of undesirable behaviors. The use of a marker also resulted in greater resistance to extinction, suggesting that conditioned reinforcers can be used effectively to maintain behavior in the absence of primary reinforcement. These findings are also likely applicable to other detection animals beyond dogs, such as rats trained to locate mines in fields at a distance from their trainer who use clickers to provide immediate conditioned reinforcement (52).

While a clicker was used as the marker in the current study, the results are likely applicable to a variety of other types of markers (e.g., whistle, tone, spoken word, or non-auditory signals) that may be preferred by some trainers, though verbal markers should be used carefully as spoken words may not be as salient of a cue due to their familiarity and inconsistency (53), but research on different types of markers is mixed and warrants further examination (11, 28, 39). Future research should also explore other applications of marker training, such as a “no reward” marker (NRM) for an incorrect response that signals the response will not be reinforced (7), and whether combining a NRM with a traditional reward-predicting marker facilitates learning; the use of other cues as a “keep going” signal for complex, long-duration behaviors that are common in military and law enforcement operations, such as tracking, search and rescue, and remote or directional guidance; as well as identifying applications where strictly marker training may not be beneficial, such as instances where it is desirable for the animal to remain in its position when being rewarded (43).

## Data Availability

The raw data supporting the conclusions of this article will be made available by the authors, without undue reservation.
